# Reply to: Uncertainty in near-term temperature evolution must not obscure assessments of climate mitigation benefits

**DOI:** 10.1038/s41467-022-31426-w

**Published:** 2022-07-14

**Authors:** Bjørn H. Samset, Jan S. Fuglestvedt, Marianne T. Lund

**Affiliations:** CICERO Center for International Climate Research, Oslo, Norway

**Keywords:** Atmospheric science, Climate change

**replying to** A. Lanson et al. *Nature Communications* 10.1038/s41467-022-31425-x (2022)

In response to our recent paper^1^ (hereafter SFL20) concerning the relation between natural variability in the climate system and the time to detect a response to emissions mitigation, Lanson et al.^2^ (hereafter L22) call for ‘a broader debate on how to best assess and communicate emerging effects of climate mitigation in the light of natural variability’. We welcome a broader debate on this aspect of global responses to climate change, attempt to reconcile our views in the following.

The scientific community should provide the best possible knowledge to policy makers and the public, while also considering their perspectives and stated needs. Applying this to the expected effects of emission mitigation in the current geophysical and political context, there are two overall approaches that can be taken. One, advocated by L22, treats quantification of this response as an exercise in detection and attribution (D&A), i.e. the statistical detection of a change relative to an expected situation and the attribution of this change to a specific cause. The other, used in SLF20 and other recent works^3–5^, focuses on detection of emergence, through observable, well communicated properties of the climate system such as global mean surface temperature (GMST)^6^. Emergence is defined as the time at which the observed signal of a change, with the influence of internal variability removed, evolves outside the range expected from this variability.

L22 rightly point out that D&A of anthropogenic climate influence in the presence of natural variability is well established^7–11^. The technique would be crucial for communication in a situation where emissions decline while GMST keeps rising due to natural variability and the inertia of the climate system. The challenge is that it requires thorough explanation to avoid being perceived as counter to the key message of climate science: That more emissions imply higher temperature.

Focusing solely on GMST evolution, as was done in SFL20, differs from this approach, by not formally utilizing decomposition of the temperature trend in terms of its underlying drivers. We emphasize that in the present situation of high emissions and a positive warming rate, mitigation of warming emissions acts to reduce future human induced climate related hazards, whether it is formally detectable or not^12–14^. When we still caution that we should curb our expectations for the near-term climate impacts from mitigation, it is because the focus on GMST as the sole indicator of progress in terms of the ambitions of the Paris Agreement has been made so strong in the media and public debate.

In line with previous arguments^15^, we therefore encourage applying both perspectives. We need to be clear on how much is known about the climate system and how we affect it through anthropogenic activities and individual drivers of climate change. We also need to be clear on the magnitude of this influence, relative to natural variability, on shorter timescales such as 10 or even 5 years.

L22 make an example of the so-called ‘global hiatus’ period, which has later been diagnosed in detail with D&A and other tools^16^, and has taught us much about energy transport through the climate system. At the time, however, communications were challenging, as GMST was the sole quantity of discussion in many fora^15^, even though the science of forcing and response was as established then as it is today.

Such a situation can easily arise again, through either temporary lower warming rates in the presence of continued emissions, or high warming in a situation of very strong mitigation. This is well known in the scientific community, but not so well established in the public debate. In fact, both combinations can be found in the simulations used in SFL20, persisting for a decade or more. See Fig. 1, where we have picked two example realizations of temperature evolution from MAGICC6^17^ combined with natural variability from the CESM Large Ensemble^18^. Through most of 2020–2030, a high-emission pathway (RCP8.5) is associated with global temperatures markedly below that of a low-emission projection (RCP2.6). For the 5-year period 2021–2025, the average difference is >0.2 °C. Both evolutions also show substantial, and statistically equivalent, multi-decadal warming trends, and approach similar warming in 2030.

**Fig. 1:**
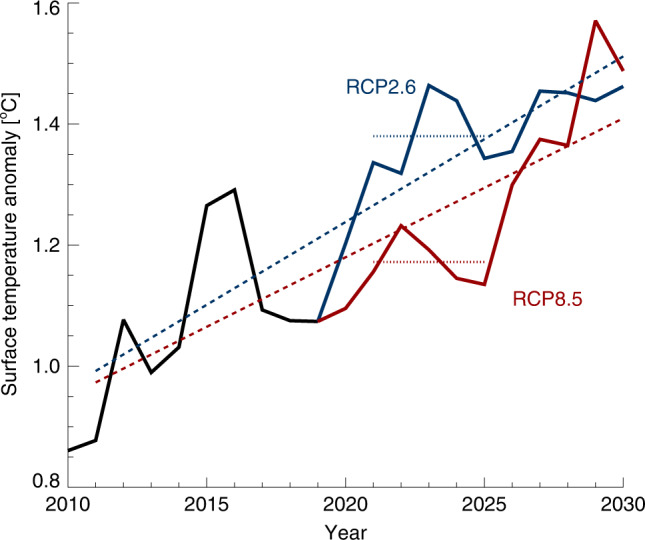
Potential evolution of global, annual mean surface air temperature (GSAT), following RCP4.5 until 2020, and then RCP2.6 (blue) or RCP8.5 (red) thereafter. The lines combine GSAT from MAGICC6 with internal variability from CESM LENS. For most of 2021–2030, the variability induces a markedly higher GSAT in the low-emission (RCP2.6) situation. Dashed lines show 20-year trends (2011–2030).

The results of Fig. 1 are not surprising, but serve as an illustration of the influence of past human emissions, and the strength of natural variability in GMST relative to the forced climate response, on such short timescales. Given that the CO_2_ emissions gap between RCP2.6 and RCP8.5 is nearly 30 GtCO_2_ in 2030^19^, the communication challenge in such a situation would lie in reconciling this vast mitigation effort with a potential perceived lack of observable results.

In addition to advancing the D&A approach, as advocated by L22, a fruitful avenue of research and communication is to consider the co-evolution of several observable quantities with (fully or partially) uncorrelated variability. The climate community has long been clear that surface temperature is only one aspects of climate change. Heightened communications focused on the evolution also of other quantities (combined with other co-benefits from emissions reductions) would reduce the reliance of the public and policy debates on this one quantity, showcasing a broader spectrum of ongoing changes. Possibilities include the rate and intensity of extreme heat and precipitation events, the diurnal temperature range over land, sea ice extent, ocean heat content, and indicators from the biosphere. These indicators will have different regional patterns and signal-to-noise ratios, and would together yield more information than GMST alone, and potentially a clearer—and earlier—emergence of an observable climate response to mitigation. L22 also refer to this possibility when they point out that 2021 seems to have had ‘outsized number of devastating extreme events despite its GMT predicted to be colder than the previous 6 years’. Here, however, we urge caution when relying on measurements from one single year—the expected rates and magnitudes of extreme events under ~1.1 °C of GMST warming are clearly not yet known. This is rather a reminder of the importance of having multiple indicators of change, such as the complementary approaches offered by emergence analyses, observations of extreme events, and more formal D&A techniques.

On the technical question raised by L22 of the choice of statistical test in SFL20, we acknowledge that the method relies on t-testing on short time series early in the evolution. It is still efficient in revealing differences between the rapid climate response to aerosol perturbations, similar to the idealized test performed by L22, and the slower evolution from e.g. CO2 emissions. Note that SLF20 also includes a consistency check with more rigorous Bayesian testing following Marotzke 2018^4^.

In conclusion, we welcome this continuation of the long-standing debate on how to communicate knowledge of the climate system and its response to emission mitigation, in the presence of both natural variability and heightened public interest. While full D&A is, and should remain, the best available technique for understanding of the ongoing changes throughout the climate system, it remains prudent to keep reminding the public, policy makers and the scientific community that it will take many years before the effects of emissions reductions will be detectable in terms of GMST alone. We may yet see a long period of high rates of surface warming, and other changes throughout the climate system, even if the crucially important efforts to rapidly reduce emissions are successful. Similarly, it remains prudent to communicate the co-benefits of rapid emissions reductions outside the influence on global surface temperatures, such as the health benefits of reduced air pollution.

## Data Availability

MAGICC6 is publicly available at live.magicc.org. CESM1 LENS simulations are available through http://www.cesm.ucar.edu/projects/community-projects/LENS/. The emission scenarios designed for SFL20 and re-used in the present reply, and the corresponding output from MAGICC6, are available through Figshare (10.6084/m9.figshare.12366335.v1).
